# Complete Profiling of Methyl-CpG-Binding Domains for Combinations of Cytosine Modifications at CpG Dinucleotides Reveals Differential Read-out in Normal and Rett-Associated States

**DOI:** 10.1038/s41598-020-61030-1

**Published:** 2020-03-04

**Authors:** Benjamin C. Buchmuller, Brinja Kosel, Daniel Summerer

**Affiliations:** Faculty of Chemistry and Chemical Biology, TU Dortmund University, Otto-Hahn-Str. 6, 44227 Dortmund, Germany

**Keywords:** DNA, DNA methylation, DNA-binding proteins

## Abstract

5-Methylcytosine (mC) exists in CpG dinucleotides of mammalian DNA and plays key roles in chromatin regulation during development and disease. As a main regulatory pathway, fully methylated CpG are recognized by methyl-CpG-binding domain (MBD) proteins that act in concert with chromatin remodelers, histone deacetylases and methyltransferases to trigger transcriptional downregulation. In turn, MBD mutations can alter CpG binding, and in case of the MBD protein MeCP2 can cause the neurological disorder Rett syndrome (RTT). An additional layer of complexity in CpG recognition is added by ten-eleven-translocation (TET) dioxygenases that oxidize mC to 5-hydroxymethyl-, 5-formyl- and 5-carboxylcytosine, giving rise to fifteen possible combinations of cytosine modifications in the two CpG strands. We report a comprehensive, comparative interaction analysis of the human MBD proteins MeCP2, MBD1, MBD2, MBD3, and MBD4 with all CpG combinations and observe individual preferences of each MBD for distinct combinations. In addition, we profile four MeCP2 RTT mutants and reveal that although interactions to methylated CpGs are similarly affected by the mutations, interactions to oxidized mC combinations are differentially affected. These findings argue for a complex interplay between local TET activity/processivity and CpG recognition by MBDs, with potential consequences for the transcriptional landscape in normal and RTT states.

## Introduction

**Figure 1:**
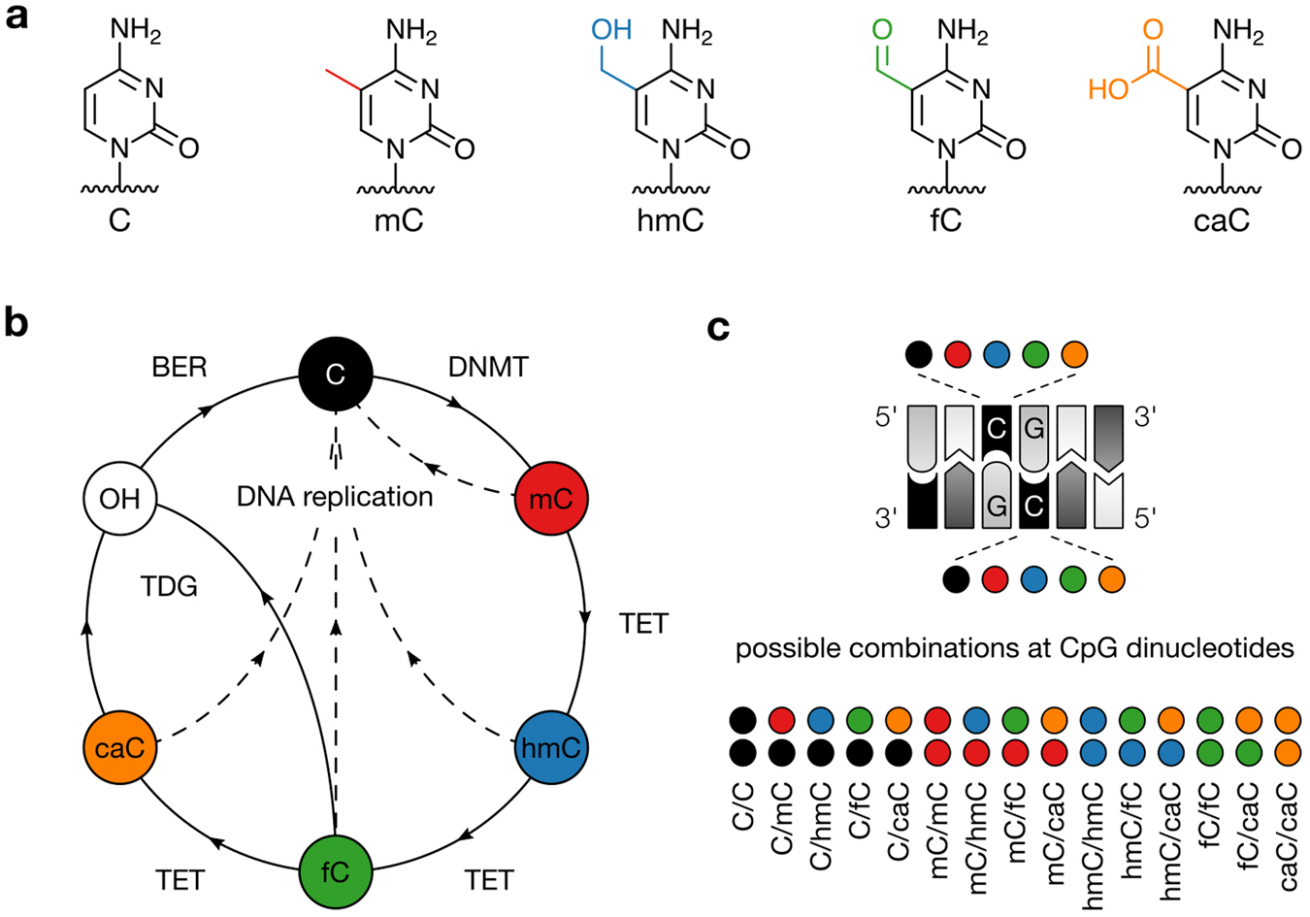
Structure, turnover, and dual-strand combinations of cytosine and its 5-modifications. (**a**) Structures of C, mC, hmC, fC, and caC. (**b**) Methylation and demethylation pathways for cytosine. (DNMT: DNA methyltransferase, TET: ten-eleven-translocation dioxygenase, TDG: thymine DNA glycosylase, BER: base excision repair, OH: abasic site). (**c**) Possible combinations of cytosine nucleobases at both strands of a CpG dinucleotide.

5-Methylcytosine (mC, Fig. 1a) is the most abundant epigenetic modification of mammalian DNA and plays important roles in differentiation, development, X-chromosome inactivation, and genomic imprinting^1^. Consequently, aberrant DNA methylation has been linked to multiple diseases including cancer^2^. mC is introduced and maintained predominantly at CpG dinucleotides by DNA methyltransferases (DNMT) and represents a stable regulatory element leading to long-term transcriptional down-regulation^1^. mC can further be oxidized by TET dioxygenases to 5-hydroxymethyl- (hmC), 5-formyl- (fC) and 5-carboxylcytosine (caC, Fig. 1a,b). Previous studies indicate that TETs can carry out stepwise oxidation of mC in a non-processive manner, giving rise to a total of fifteen theoretical combinations of cytosine modifications on both strands of a CpG dinucleotide^3–5^ (Fig. 1c). This combinatorial space can be further modulated by base excision repair (BER) of fC and caC, and the presence or absence of post-replicative maintenance methylation (Fig. 1b)^6–10^.

Each combination of cytosine modifications at CpGs constitutes a chemically distinct interaction surface that may be interpreted differently by proteins interacting with both strands of the CpG dinucleotide. Indeed, differential interactions of multiple key nuclear proteins with oxidized mCs have been reported^11–19^.

Proteins containing a methyl-CpG-binding domain (MBD) are the central readers of methylated CpG dinucleotides and interact with both DNA strands^20^. The “core” MBD family proteins include MeCP2 as well as MBD1–4, sharing a conserved MBD. Additional domains translate CpG methylation into repressive chromatin states by interactions with histone lysine methyltransferases and demethylases, deacetylases, DNMT, chromatin remodeling complexes, and others^20^.

MeCP2^21^ plays particularly important roles in the brain, indicated by its exceptionally high expression levels that approach the ones of histones^22^. Intriguingly, oxidized mCs are particularly abundant in brain and embryonic stem cells (ESC), where hmC levels can be stable^23^ and reach up to 20–40% of all mCs^24^. Similar observations have been made for fC levels at certain ESC positions^25^. Mutations in the MBD of MeCP2 are a causative of Rett syndrome (RTT) and often characterized by altered binding to methylated CpG^26–28^. Hence, a deeper understanding of the direct interactions of MBDs, including MeCP2 RTT mutants, with oxidized mC combinations at CpGs is of particular interest, since they may translate into altered genomic distributions of MBD occupancy and transcriptional activity in normal and RTT-associated states.

Interactions of several full-length MBD proteins and isolated MBDs from different organisms with differentially modified CpGs have been evaluated^29–34^. Although these studies delivered highly valuable insights into the interplay of individual MBDs and particular combinations of cytosine modifications, they are incomplete in view of the tested MBD-CpG combinations and allow only limited comparisons. The latter is due to the use of either full-length or isolated MBDs from different organisms, different expression construct designs, as well as different binding conditions and target DNA sequences. It is further unknown, how different RTT-associated MBD mutants of MeCP2 interact with combinations of hmC and higher oxidized mCs.

Here, we report complete, comparative interaction profiles of human MeCP2 and MBD1–4 with all fifteen combinations of modified cytosine nucleobases at CpGs, revealing individual preferences of each MBD for distinct combinations. In addition, we profile four MeCP2 RTT mutants and report that though binding to methylated CpGs is similarly reduced by the mutations, their interaction to frequent oxidized mC combinations are differentially affected. These findings argue for a more complex interplay between TET activity and MBD than previously thought that may translate into altered genomic landscapes of MBD occupancy and transcriptional activity.

## Results

**Figure 2:**
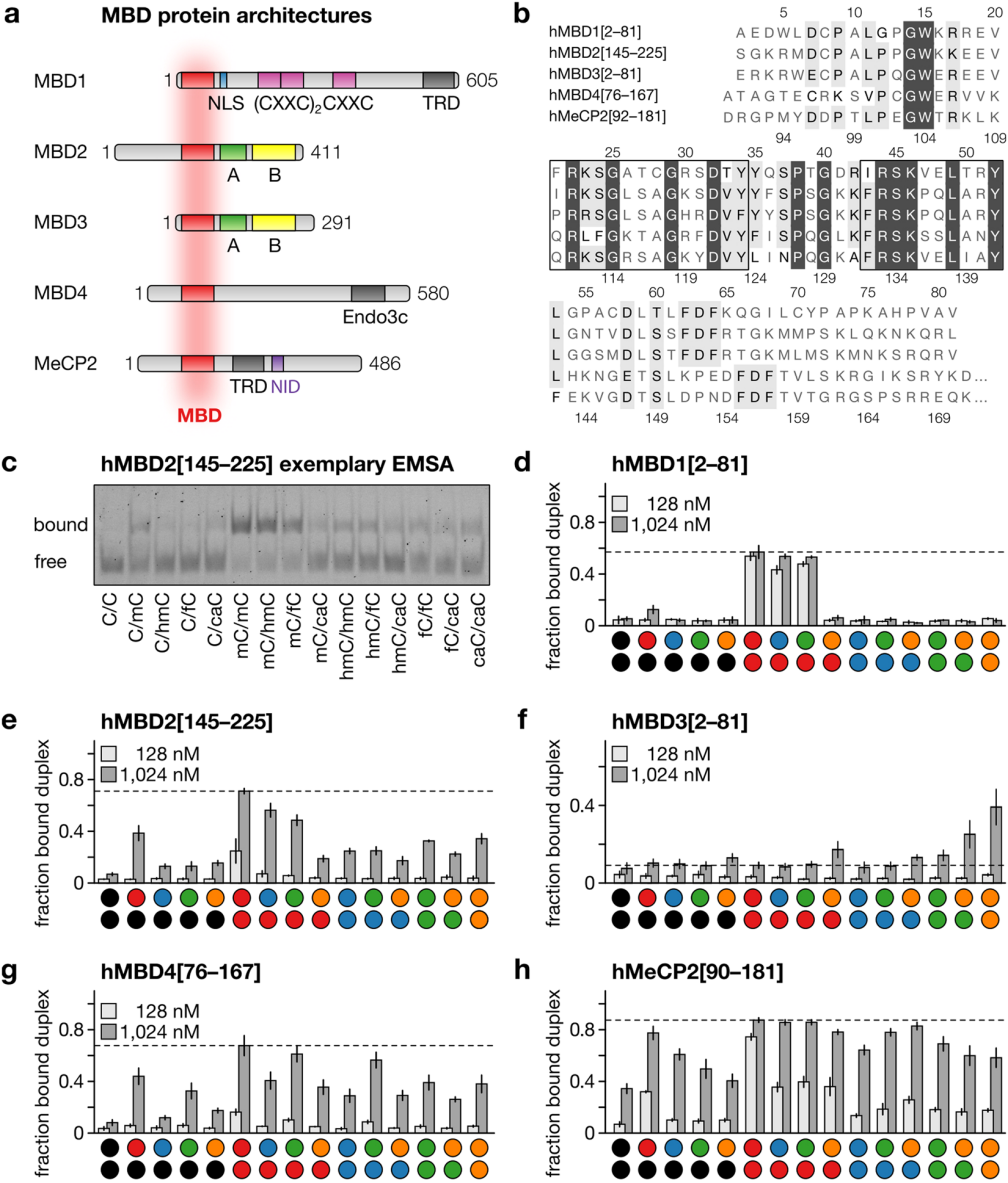
MBDs and DNA recognition. (**a**) Protein architecture of five MBD family proteins. (**b**) Amino acid sequence of human MBD domains used in this study. Identical residues in dark gray and residues with similar properties in light gray; residues in vicinity to the DNA duplex are framed. (**c**) Representative EMSA of hMBD2[145–225]. (**d**–**h**) Bar diagrams of the fraction of MBD-bound DNA duplex observed in EMSA for MBDs and nucleobase combinations as indicated. Data are means ± SEM from three independent experiments (see SI for full data).

We recombinantly expressed the MBDs of the five human MBD family proteins hMBD1–4 and hMeCP2 (Fig. 2a), which all adopt a highly similar three-dimensional fold and share 50–60% of polypeptide sequence identity, especially at the residues interacting with the DNA double-strand (Fig. 2b)^20^. To test whether this would result in a more similar or dissimilar read-out of differentially modified CpG dinucleotides, we designed target oligodeoxynucleotide duplexes as 24-mers containing a single, central CpG in an oligo-dA/dT context. We chose this reductionistic approach since sequence contexts varied among previous studies and context preferences have been described for MBDs. Our target design reduces the number of CpG combinations from 25 (with context) to only 15, and offers profiling of CpG interactions without potential context preferences.

To obtain data within the dynamic range of complex formation, we evaluated the binding of each MBD at one higher and one intermediate protein concentration with all possible cytosine nucleobase combinations at the CpG using electromobility shift assays (EMSA, Fig. 2c–h).

In agreement with previous studies using related constructs, hMBD1 (Fig. 2d, Supplementary Fig. 1) bound mC/mC and mC/hmC strongly, C/mC with markedly reduced affinity, and C/C, C/hmC and hmC/hmC not at all^35,36^.

However, our study additionally revealed that mC/fC, but not mC/caC, is a CpG combination recognized by hMBD1. Strikingly, none of the other combinations was bound, indicating a strict dependence of hMBD1 on the presence of at least one mC. The presence of any combination of oxidized mC at both positions or in combination with C or caC abolished binding.

In contrast, hMBD2 interacts with multiple combinations containing one or even two modified cytosines (including caC, Fig. 2e, Supplementary Fig. 2). If one of these modifications was mC, binding of hMBD2 was stronger (except for mC/caC), and strongest for mC/mC. These observations agree with a study on murine Mbd2 in complex with transcriptional repressor p66α, which reports affinities as: mC/mC > C/mC, C/hmC > hmC/hmC^35^.

Next, we evaluated hMBD3, a key component of the Mi-2/NuRD nucleosome remodeling and deacetylase complex. MBD3 shares 70% amino acid sequence similarity with MBD2^20^, but contains the mutations K30H and Y34F (Fig. 2b, Supplementary Fig. 3) that reduce the binding to methylated CpGs^37^. The murine orthologous protein has previously been shown to interact with mC/mC and other combinations involving C, mC and hmC only very weakly^35^. Indeed, we also observed overall low binding to these combinations, but also to most other previously not evaluated combinations. However, in our assay, binding of hMBD3 was slightly less reduced in presence of a caC nucleobase in a CpG, preferentially when paired with a second caC or an fC (Fig. 2f).

The MBD of hMBD4, of which the full-length protein exerts DNA glycosylase activity involved in base excision repair^20^, is known to preferentially bind mC/mC, but with comparably low selectivity. Combinations mC/hmC and mC/fC are bound with similar affinity, higher than hmC/hmC and mC/caC^36^. Our binding data are in agreement with these findings, with the exception that we observed higher binding to mC/fC than to mC/hmC (Fig. 2g, Supplementary Fig. 4). Moreover, our extended interaction profiles revealed hmC/fC as a new preferred combination. The same was true for C/fC, fC/fC and caC/caC, albeit with lower affinity.

hMeCP2 exhibited the highest overall affinity of the MBDs and a clearly pronounced mC/mC selectivity (Fig. 2h, Supplementary Fig. 5). The second highest affinities were observed for any combination with mC, including (and in stark contrast to MBD1 and MBD2) mC/caC. The presence of C was generally causing particularly low affinities compared to other modified cytosines.

Taken together, MBDs showed markedly different selectivity profiles for differentially modified CpGs despite their high degree of sequence conservation, particularly at residues interacting with the CpG. We were therefore wondering how RTT-associated single amino acid substitutions in MeCP2 would affect the selectivity profile of its MBD.

**Figure 3:**
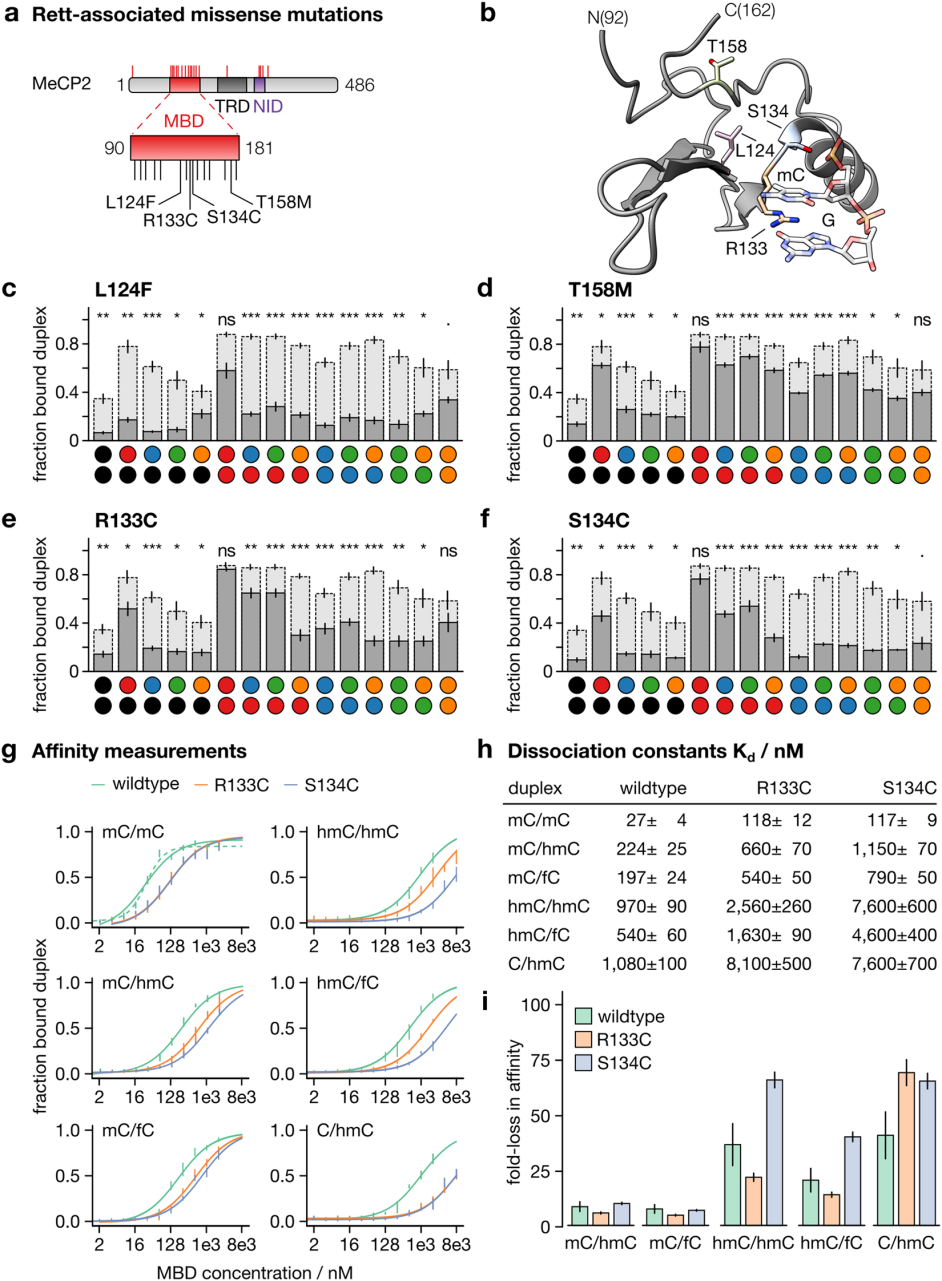
Interaction of MeCP2 Rett mutants. (**a**) Protein architecture of MeCP2 and positions of studied mutations. (**b**) Rett mutations highlighted on MeCP2 in complex with mC/mC containing DNA (PDB entry 3C2I^49^. Prepared with UCSF ChimeraX^50^. (**c**–**f**) Bar diagrams of the fraction of MBD-bound DNA duplex observed in EMSA for MBDs at 1,024 nM and nucleobase combinations as indicated in dark grey; data for wildtype hMeCP2[90–181] underlaid in light gray for comparison. Data are means ± SEM from three independent experiments (see SI for full data). (**g**) K_d_ log diagrams of wildtype and Rett mutants. (**h**) Table of K_d_ values obtained from measurements in (**g**). (**i**) Fold-loss in affinity (on basis of K_d_) for wildtype hMeCP2 and Rett mutants with respect to their affinity for mC/mC; mean ± SEM.

Indeed, about half of the RTT-causing mutations of hMeCP2 cluster in its MBD domain (Fig. 3a) and are often characterized by altered binding to methylated CpGs (Fig. 3b)^38^. It is however poorly understood how these mutations may lead to differential interpretation of combinations involving oxidized mC nucleobases. To this end, we evaluated the frequently occurring mutants L124F, T158M, R133C and S134C^38^ using EMSA (Fig. 3c–f, Supplementary Figs. 6–10).

Overall, the mutants exhibited lower binding than wildtype hMeCP2, and retained overall mC/mC selectivity. Moreover, combinations containing an mC were typically bound comparatively strong for T158M, R1333C and S134C, with differences in the individual selectivities. Particularly, mC/caC was bound less by the R133C and S134C mutants as compared to wildtype hMeCP2 and T158M. In contrast, L124F showed overall weak binding of mC-containing combinations other then mC/mC (Fig. 3e,f).

Moreover, whereas T158M, R133C and S134C exhibited comparable selectivity profiles as wildtype hMeCP2 for the five C-containing combinations (with C/mC as preferred combination), L124F slightly preferred C/caC over C/mC. Similarly, we observed a slightly preferred interaction with caC/caC compared to other higher oxidized combinations for L124F and R133C (Fig. 3c,e).

A particularly noteworthy difference was the seemingly higher loss in affinity for hmC/hmC of the S134C mutant as compared to its loss in affinity for mC/mC or mC/hmC, because these lower oxidized combinations are likely to occur most frequently in brain cells^24^. We therefore measured the K_d_ values of wildtype hMeCP2 and the R133C and S134C mutants for the lower oxidized combinations mC/hmC, hmC/hmC, mC/fC and hmC/fC representing the initial oxidation products of TET activity. We further included the combination C/hmC in our analysis, an expected frequent product of the “active modification-passive dilution” demethylation pathway (Fig. 3g,h, Supplementary Figs. 11–12)^24^. Both mutants exhibited identical, ~4-fold reduced affinity for the cognate mC/mC combination (Fig. 3i). However, there was a striking difference between R133C and S134C in binding to combinations containing an oxidized mC.

Specifically, affinities of S134C compared to R133C were reduced ~3-fold for hmC/hmC and hmC/fC, i.e. comparable to the reduction of mC/mC affinity observed for both mutants compared to wildtype hMeCP2. The only exception of this discrepancy was the non-mC containing combination C/hmC, where again almost identical K_d_ were observed.

## Discussion

The here established structure-function-relationships reveal markedly different abilities of the human core family MBDs to discriminate between cognate mC/mC CpG and their TET-generated oxidation products. Previous studies have reported mC/mC selectivity for the four functional MBDs (i.e. excluding MBD3) in the context of several oxidized mC combinations. The most comprehensive study covered the MBD domains of all five core MBD family proteins and all combinations of C, mC and hmC in both CpG strands, though mixed comparisons with MBDs from either mouse or human were made^35^. hmC generally led to a reduced affinity of MBDs, arguing for a model in that mC oxidation primarily alters the MBD landscape by reducing occupancy at oxidized sites. Also, the interactions of hMBD1 and hMBD4 with DNA containing all three oxidized mCs in combination with mC have been characterized^36^.

Our extended studies reveal that this overall selectivity is retained in the context of all fifteen CpG combinations and all functional MBDs.

Our study reveals individual preferences of MBDs for several oxidized combinations within previously uncharacterized interactions, being somewhat in contrast with the comparably consistent selectivity for fully versus hemi- or non-methylated CpG. Overall, the presence of at least one mC typically led to high affinities, with reductions for the second cytosine, if oxidized. This argues for a potential release of MBDs at pre-exisiting genomic mC/mC sites upon TET oxidation. However, compared to the “classic” off-target combinations C/C and in many cases also mC/C, the oxidized CpGs mC/hmC, mC/fC and mC/caC were recognized with higher affinities (in case of hMBD4 also hmC/fC), and with pronounced individual preferences of the individual MBDs.

These combinations may therefore act as attenuated recruitment signals that can be differentially read by the four MBDs and result in differential biological outputs. For example, given the competition between TETs and MBDs for CpG^39,40^, MBDs may differentially modulate the processivity of TETs at such sites. Similarly, positive recruitment processes between the two proteins may be differentially affected^39,41^.

High expression of hMeCP2 and high levels of oxidized mCs are hallmarks of brain cells, and mutations of the hMeCP2 MBD with reduced binding to methylated CpG are a causative of RTT. Indeed, reduced affinities have been described in a study covering interactions of hMeCP2 with all oxidized mC combinations^42^. We report the first comprehensive profiles of RTT mutants for all oxidized mC combinations at CpG. These reveal overall reduced binding of the studied mutants, albeit with marked differences in respect to oxidized mC combinations. In particular, mutations R133C and S134C have identical effects on binding to mC/mC, whereas the latter mutation affects binding to oxidized mC combinations much stronger, suggesting that individual RTT mutations may result in different genomic MeCP2 distributions. Overall, it should be noted that CpG sequence context preferences have been described for MeCP2 and several other MBDs^20^, which may further complicate the picture.

Overall, our study provides comprehensive, comparable interaction profiles of MBDs with individual oxidized mC combinations at both strands of CpG, and thus refined insights into how TET-mediated mC oxidation may modulate landscapes of MBD occupancy and transcriptional activity in normal and RTT-associated states.

## Methods

### Plasmids

For cloning of MBD expression plasmids (Supplementary Table 1), pET-21d(+) (Merck, Darmstadt, Germany) was digested with *Xho*I and *Nco*I (New England Biolabs) to replace the T7 tag by Gibson assembly^43^ with the synthetic Z domain of staphylococcal protein A (SpA)^44^. This was amplified from an accessory plasmid using primers o2872/o2873, introducing the start codon ATG along with a factor Xa and a TEV recognition and cleavage site. The resulting vector pBeB1380 allowed expression of N-terminal SpA(Z) fusion proteins with a non-cleavable C-terminal 6xHis tag. pBeB1380 was linearized with *Xho*I, and the codon-optimized sequences of the human MBD protein domains obtained as gBlocks (Integrated DNA Technologies, Supplementary Table 2) were amplified and introduced by Gibson assembly. Due to the repetitive sequence encoding the 6xHis tag, this assembly resulted in 8xHis-tagged fusion proteins. The consensus coding sequences (CCDS) of the human MBD proteins (Supplementary Table 3) were obtained from the CCDS project^45^, which were identifiers 59318.1 for hMBD1[2–81] (pBeB1389), 11953.1 for hMBD2[146–225] (pBeB1390), 12072.1 for hMBD3[2–81] (pBeB1391), 3058.1 for hMBD4[76–167] (pBeB1392), and 14741.1 for hMeCP2[90–181] (pBeB1393). The MBD domain within the coding sequences was identified by alignment versus Pfam PF01429 and flanked with about 5 to 15 additional amino acids at the N- and C-terminus of the domain, respectively. The Rett-associated mutations in hMeCp2 were introduced using QuikChange site-directed mutagenesis (Agilent; Supplementary Table 2). For a sample plasmid map, see Supplementary Fig. 13.

### Expression and purification of MBDs

Similar to a protocol of Free *et al*.^29,46^, expression plasmids were transformed into *E. coli* BL21-Gold(DE3) (Agilent), and fresh overnight cultures of single clones were diluted to an optical density (OD_600_) of 0.05 in 30 mL LB-Miller broth supplemented with 50 µg/mL carbenicillin, 1 mM MgCl_2_ and 1 mM ZnSO_4_^35^. Cultures were grown at 37 °C (220 rpm) to an OD_600_ of 0.5–0.6, briefly chilled on ice, and then induced by supplying 1 mM isopropyl β-d-1-thiogalactopyranoside (IPTG). Cultures were incubated at 25 °C (150 rpm) for at least 6 h or overnight, and cells were harvested and washed once by resuspension in 0.25 vol ice-cold 20 mM Tris-HCl (pH = 8.0). Pellets were resuspended in 2 mL binding buffer (20 mM Tris-HCl, 250 mM NaCl, 10% glycerol, adjusted to pH = 8.0, supplemented with 10 mM 2-mercaptoethanol, 5 mM imidazole and 0.1% Triton X-100), and sonicated in a Bioruptor Pico (Diagenode) at 4 °C using 3 × 4 cycles (30 s pulse of 20–60 kHz, 25–200 W and 30 s rest). Suspensions were treated with 0.1 mg/mL lysozyme (Merck) and 10 U/mL DNase I (New England Biolabs) overnight. After centrifugation at 14,000 × *g* for 20 min at 4 °C, the cleared supernatants were retained, diluted with 1 vol binding buffer, mixed with 450 µL 50% Ni-nitriloacetic acid (NTA) HisPur agarose resin (ThermoFisher), and incubated at 4 °C for 2 h. The resins were washed 2 x with 1 mL binding buffer containing 90 mM imidazole (20 min at 4 °C) and the fusion proteins were eluted in 2 × 0.2 mL and 1 × 0.4 mL binding buffer with 500 mM imidazole (10 min at 4 °C). Fractions judged to be >90% pure (SDS PAGE) were combined and dialyzed against 3 × 15 mL 20 mM HEPES, 100 mM NaCl, 10% glycerol, adjusted to pH = 7.3, and 0.1% Triton X-100 in Slide-A-Lyzer MINI devices (3.5 kDa MWCO, ThermoFisher). An additional 1:2–1:5 dilution is recommended when scaling up this procedure to avoid precipitation during dialysis. The protein concentrations were determined with a BCA assay (ThermoFisher) and the proteins stocked at 15 µM after snap freezing in liquid nitrogen, at −80 °C (stable for several months). Typically, 3–4 pmol SpA-MBD fusion protein are obtained per mL culture. The SpA tag can be efficiently removed with 0.25 µM TEV protease at 4 °C overnight. Uncleaved or cleaved SpA tag and the TEV protease do not interfere with oligodeoxynucleotide binding (Supplementary Fig. 14). However, it has been noted that prolonged storage of the tag-free MBDs can result in spontaneous precipitation.

### Electrophoretic mobility shift assays

The 24-mer oligodeoxynucleotide (ODNs, Supplementary Tables 4–5) pairs were combined at 1.5 µM of the labeled strand and 1.8 µM of the unlabeled strand to ensure complete duplex formation of the labeled strand. We incubated this mix in rudimentary EMSA buffer^46^ (20 mM HEPES, 30 mM KCl, 1 mM EDTA, 10 mM (NH_4_)_2_SO_4_, pH = 7.3) at 95 °C for 5 min, slowly brought it to room temperature in a water bath for duplex formation, and subsequently diluted it to 30 nM with respect to the labeled strand. The non-specific binding trap duplex was prepared by annealing a 24-mer poly(dA) with a 24-mer poly(dT) at equimolar ratios of 50 µM. EMSA were carried out according to a well-established protocol^29,34,42,46^. In brief, purified MBDs were diluted to 0, 2, 4, 8, 16, 32, 64, 128, 256, 512, and 1,024 nM in dialysis buffer with 0.1 mg/mL BSA (New England Biolabs) and incubated with 2 nM labeled duplex and 50 ng/µL poly(dA)·poly(dT) in EMSA buffer containing 1 mM dithiothreitol and 0.2% Tween 20 in a final volume of 15 µL. The binding was allowed to equilibrate for 20 min at room temperature before 3 µL of a 6x loading dye (1.5 x TBE, pH = 7.5, 40% glycerol, 70 pg/mL bromophenol blue) were added on ice. These samples (10 µL) were loaded on pre-run 0.25 x TBE, 12% polyacrylamide gels and run at 240 V for 45 min at 4 °C in Mini-PROTEAN vertical electrophoresis cells (Bio-Rad). Gels were recorded on a Typhoon FLA-9500 laser scanner (GE Healthcare) equipped with a 473 nm laser and a 510 LP filter at 700–800 V PMT amplification without over-exposure. The fraction of bound duplex was determined using ImageQuant TL v8.1 1D Gel Analysis (GE Healthcare) applying rubber band background subtraction and manual peak detection with approximately equal peak areas across all lanes.

### Data analysis and K_d_ determinations

All data was curated and analyzed with R v3.6.0. Given a single ligand binding site of the MBD protein domain and the absence of additional intramolecular interactions, the relationship between the total concentration of labeled duplex [L]_0_, added to the reaction, the total concentration of MBD [R]_0_, the fraction of bound ligand [RL]/[L]_0_ at equilibrium and the dissociation constant K_d_ follows the quadratic equation^47^.

1 $${[{\rm{RL}}]}^{2}-[{\rm{RL}}]\,({[{\rm{R}}]}_{0}+{[{\rm{L}}]}_{0}+{{\rm{K}}}_{{\rm{d}}})+{[{\rm{R}}]}_{0}{[{\rm{L}}]}_{0}=0$$

which has been used to determine the K_d_ of MBD protein domains from fractional binding by Khrapunov *et al*.^34^ as well as from free ligand, [L] = [L]_0_ − [RL], by Yang *et al*.^42^. Here, we use the solution for the experimentally observed fraction of bound ligand, [RL]/[L]_0_, which is

2 $$[{\rm{RL}}]/{[{\rm{L}}]}_{0}={({[{\rm{R}}]}_{0}+{[{\rm{L}}]}_{0}+{{\rm{K}}}_{{\rm{d}}}-[([{\rm{R}}]}_{0}+{[{\rm{L}}]}_{0}+{{\rm{K}}}_{{\rm{d}}}{)}^{2}-4{[{\rm{R}}]}_{0}{{[{\rm{L}}]}_{0}}^{1/2})/(2{[{\rm{L}}]}_{0})$$

to determine the K_d_ by non-linear curve fitting using the Levenberg-Marquardt algorithm. We propagated the uncertainty associated with the K_d_ estimates to the derived fold-changes according to the basic equation for error propagation^48^.

## Supplementary information

Supplementary information.

## Data Availability

The datasets generated and analysed during the current study are available from the corresponding author on reasonable request.
