# Supplementary material for: Complete Profiling of Methyl-CpG-Binding Domains for Combinations of Cytosine Modifications at CpG Dinucleotides Reveals Differential Read-out in Normal and Rett-Associated States
Source: Sci Rep. 2020 Mar 4;10:4053. doi: 10.1038/s41598-020-61030-1 (PMC7055227; doi:10.1038/s41598-020-61030-1)
Supplement: Supplementary file 1 — Supplementary information. [file 41598_2020_61030_MOESM1_ESM.pdf]

Supporting Online Information

Complete Profiling of Methyl-CpG-Binding Domains for Combinations of Cytosine Modifications at CpG Dinucleotides Reveals Differential Read-out in Normal and Rett-Associated States

Benjamin C Buchmuller[a], Brinja Kosel[a] and Daniel Summerer[a]*

---

[a]Department of Chemistry and Chemical Biology

Technical University of Dortmund

CP-02-143

Otto-Hahn-Str. 4a

44227 Dortmund

*to whom correspondence should be addressed: Daniel.Summerer@tu-dortmund.de

Table of Contents

Table of Contents 2

Supplementary Figures 3

Supplementary Figure 1. Selectivity measurements for human MBD1[2–81]........ 3

Supplementary Figure 2. Selectivity measurements for human MBD2[145–225]........ 4

Supplementary Figure 3. Selectivity measurements for human MBD3[2–81]........ 5

Supplementary Figure 4. Selectivity measurements for human MBD4[76–167]........ 6

Supplementary Figure 5. Selectivity measurements for human MeCP2[90–181]........ 7

Supplementary Figure 6. Selectivity measurements for human MeCP2[90–181]L124F. 8

Supplementary Figure 7. Selectivity measurements for human MeCP2[90–181]R133C. 9

Supplementary Figure 8. Selectivity measurements for human MeCP2[90–181]S134C........ 10

Supplementary Figure 9. Selectivity measurements for human MeCP2[90–181]T158M. 11

Supplementary Figure 10. Altered selectivity profiles of MeCP2 Rett mutants at low protein concentration........ 12

Supplementary Figure 11. Affinity measurements of MeCP2 Rett mutants (Representative EMSA gel images)........ 13

Supplementary Figure 12. Affinity measurements of MeCP2 Rett mutants (Fitted curves)........ 14

Supplementary Figure 13. Sample plasmid map of pBeB1380 with hMBD1[2–81]........ 13

Supplementary Figure 14. Effect of TEV cleavage on SpA(Z)-MBD fusion proteins........ 16

Supplementary Tables........ 17

Supplementary Table 1. Plasmids........ 17

Supplementary Table 2. Oligodeoxynucleotides for plasmid construction. 17

Supplementary Table 3. MBD protein sequences. 19

Supplementary Table 4. Oligodeoxynucleotides (ODNs) for EMSA........ 20

Supplementary Table 5. ODN pairs for EMSA. 21

Supplementary Table 6. Fit statistics. 22

Supplementary Figures

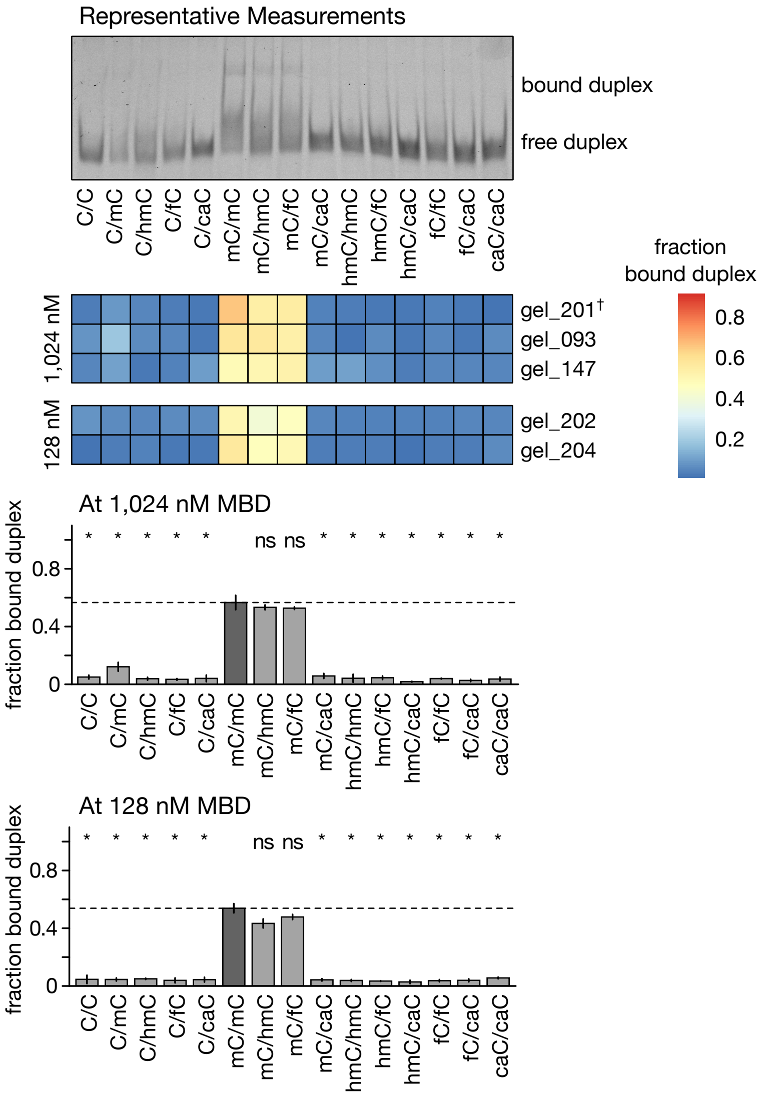

Supplementary Figure 1. Selectivity measurements for human MBD1[2–81]. A representative EMSA gel image at 1,024 nM MBD protein concentration is shown in the top panel; representative single measurements of the fraction of bound labelled DNA duplex are displayed as a heatmap in the mid panels († indicates the EMSA gel image shown) and summarized (mean) as bar graphs in the lower panels at high and low MBD protein concentration. Error bars indicate standard error of the mean with two-sided Student's *t*-test against the fraction of bound mC/mC duplex (dark gray); false-discovery rate was controlled using the Benjamini-Hochberg procedure to correct the *p*-values for multiple comparisons (ns: *p* in (0.1, 1], . (0.05, 0.1], * (0.01, 0.05], ** (0.001, 0.01], *** [0, 0.001]).

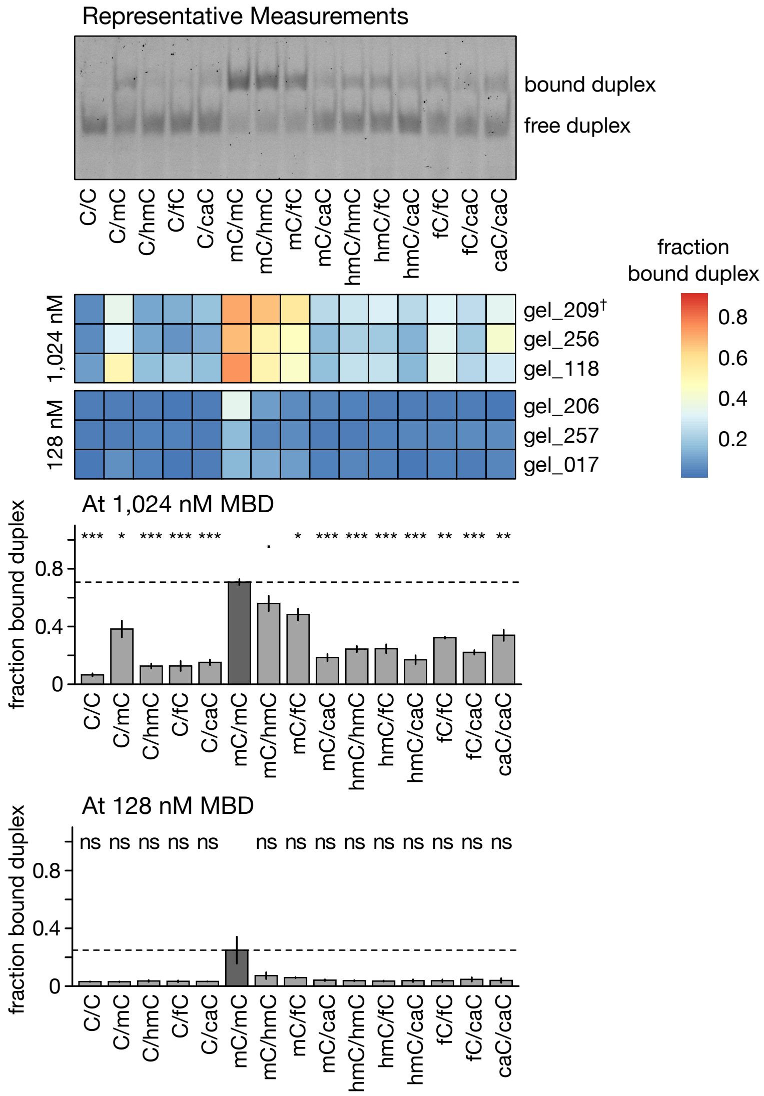

Supplementary Figure 2. Selectivity measurements for human MBD2[145–225]. All details see Supplementary Figure 1.

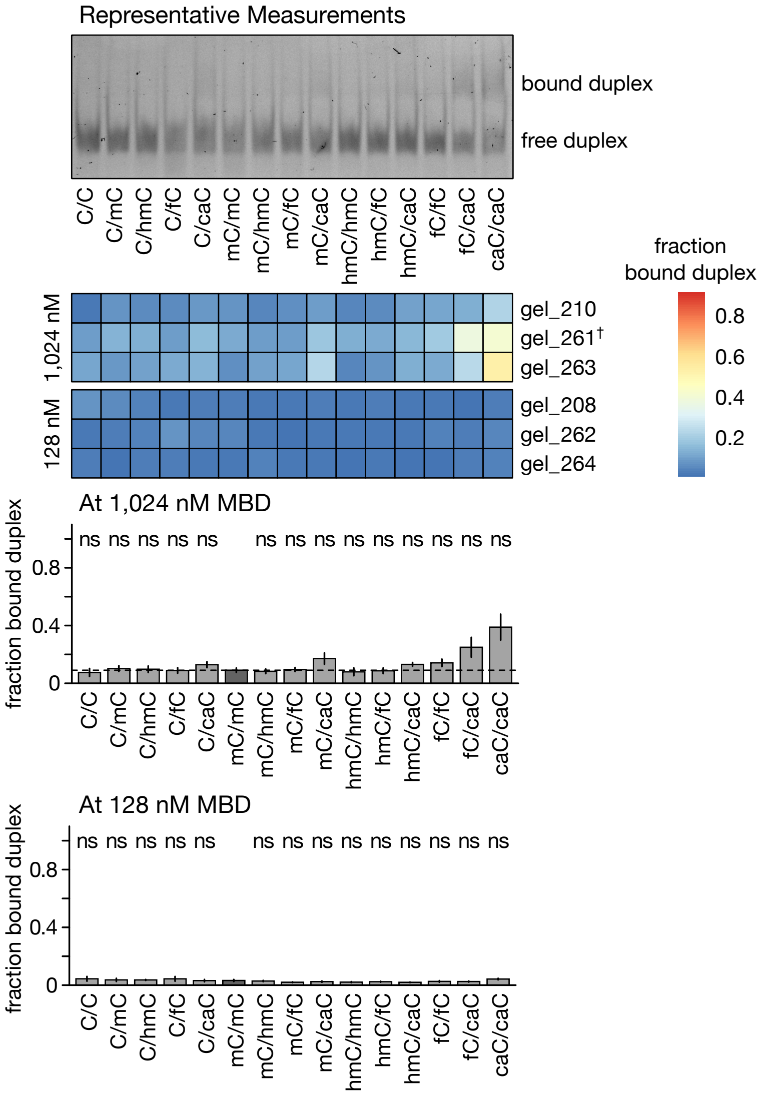

Supplementary Figure 3. Selectivity measurements for human MBD3[2–81]. All details see Supplementary Figure 1.

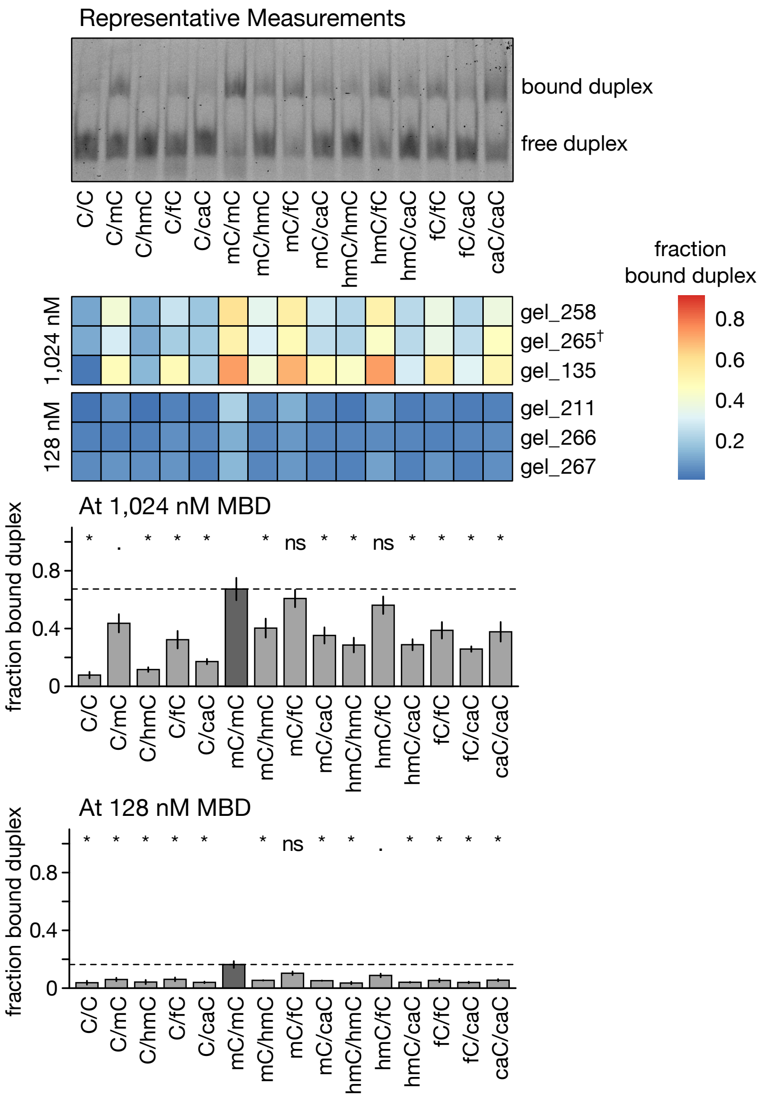

Supplementary Figure 4. Selectivity measurements for human MBD4[76–167]. All details see Supplementary Figure 1.

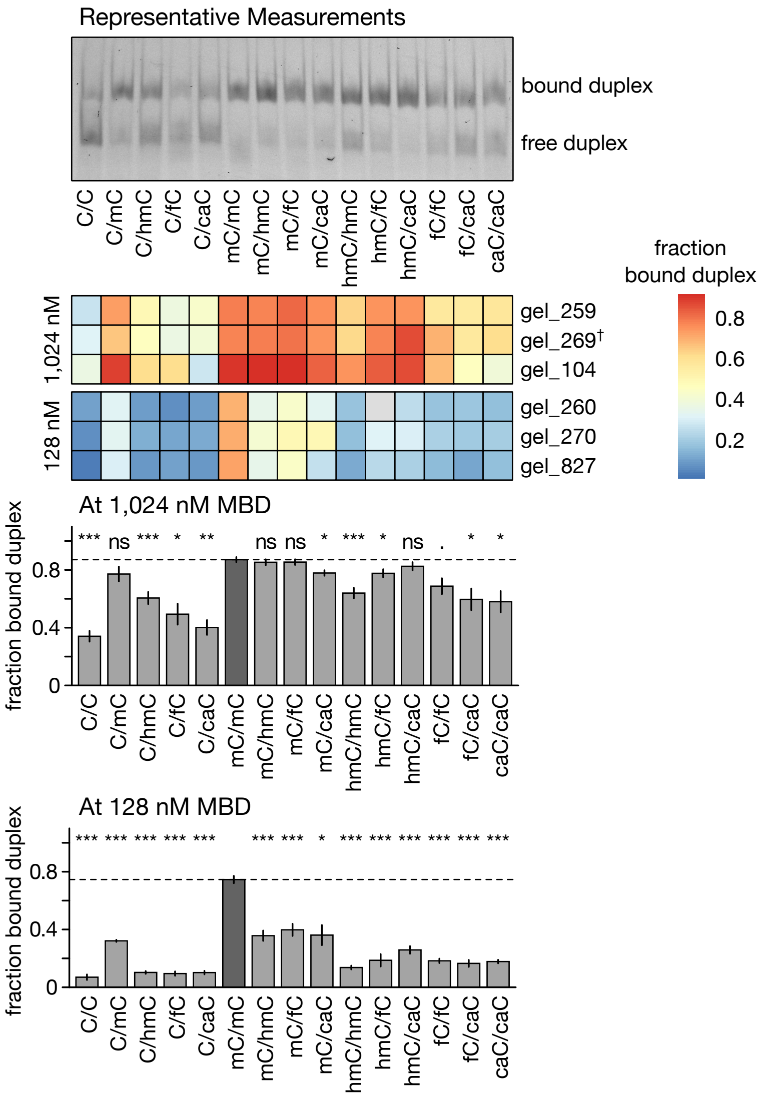

Supplementary Figure 5. Selectivity measurements for human MeCP2[90–181]. All details see Supplementary Figure 1.

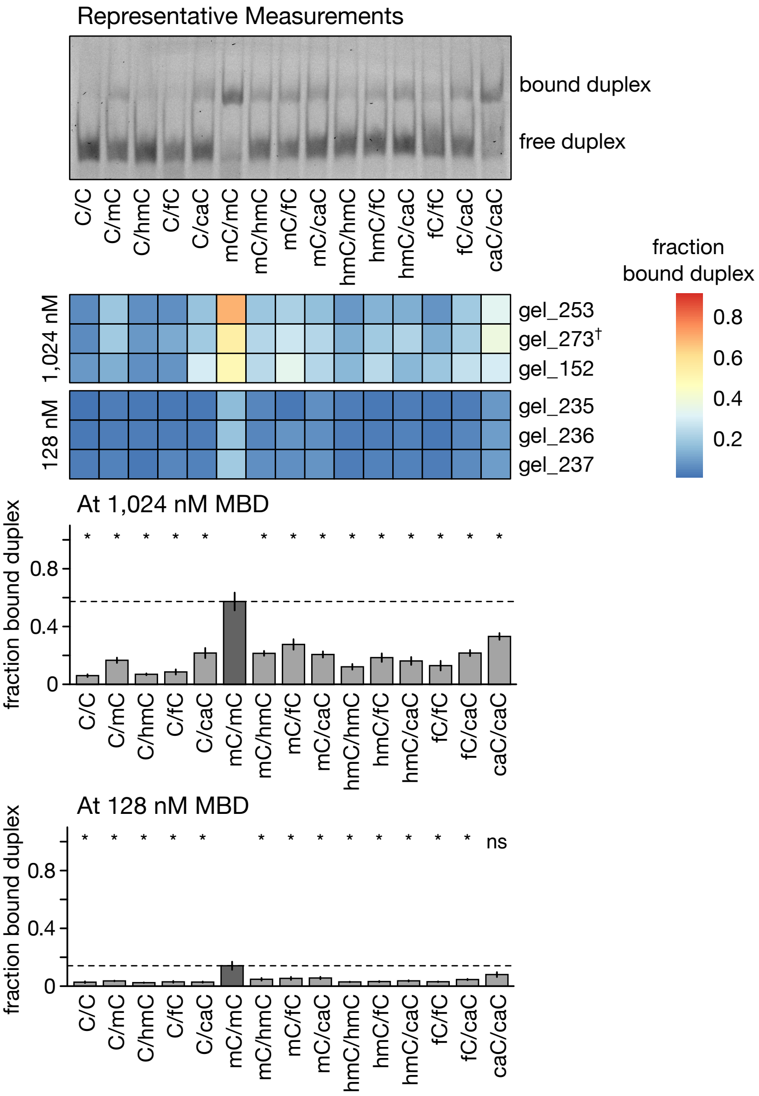

Supplementary Figure 6. Selectivity measurements for human MeCP2[90–181]L124F. All details see Supplementary Figure 1.

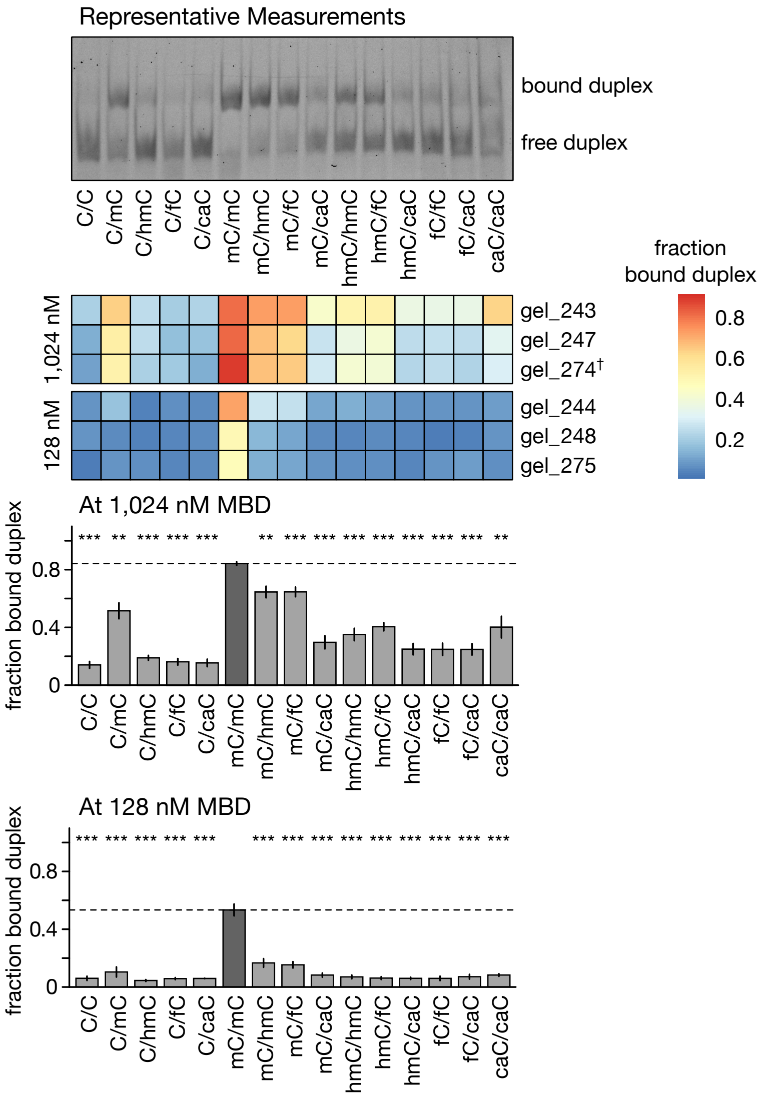

Supplementary Figure 7. Selectivity measurements for human MeCP2[90–181]R133C. All details see Supplementary Figure 1.

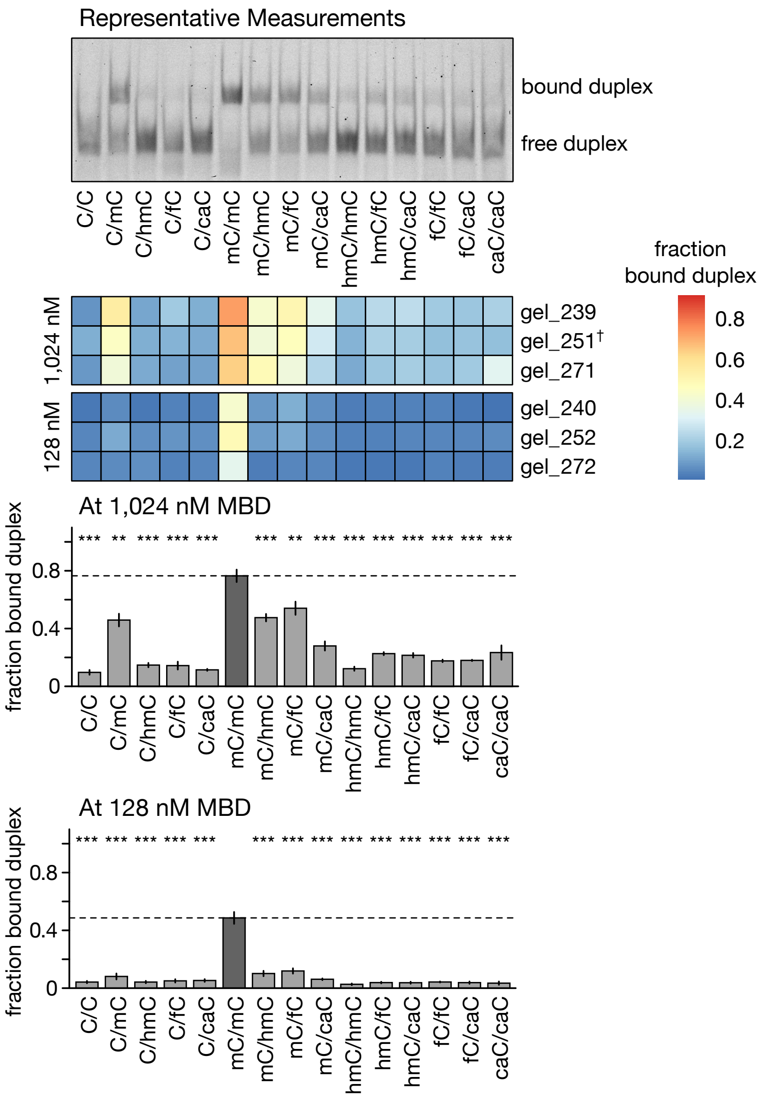

Supplementary Figure 8. Selectivity measurements for human MeCP2[90–181]S134C. All details see Supplementary Figure 1.

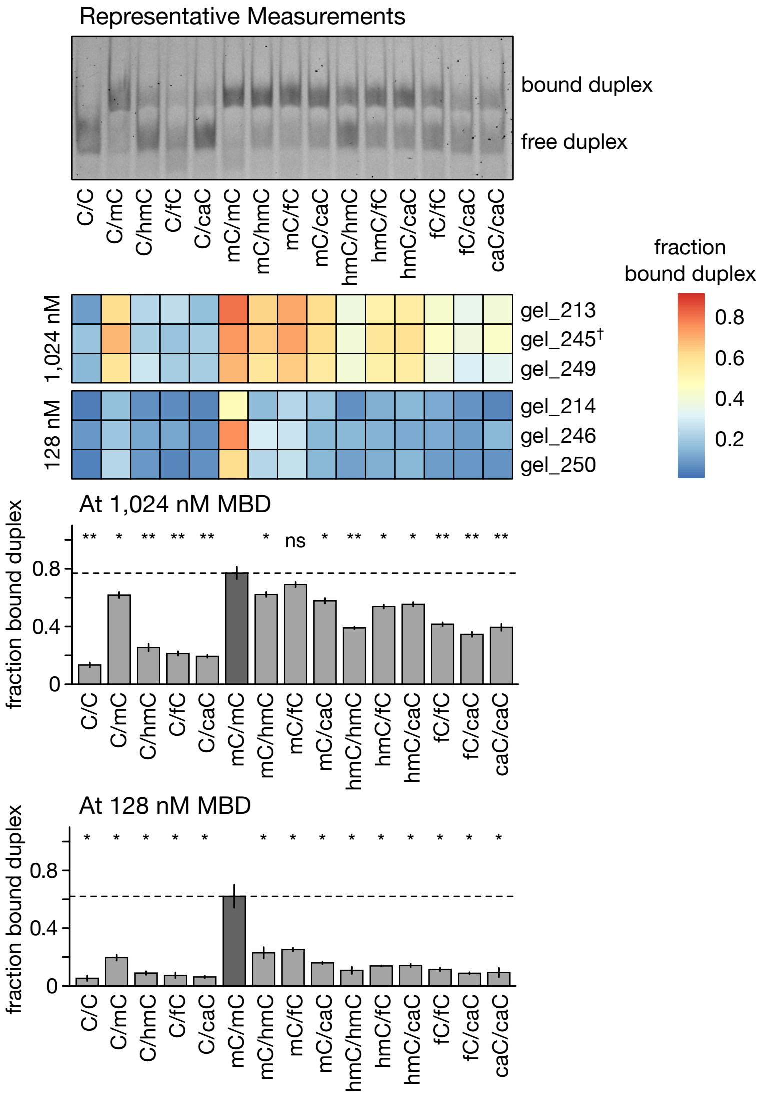

Supplementary Figure 9. Selectivity measurements for human MeCP2[90–181]T158M. All details see Supplementary Figure 1.

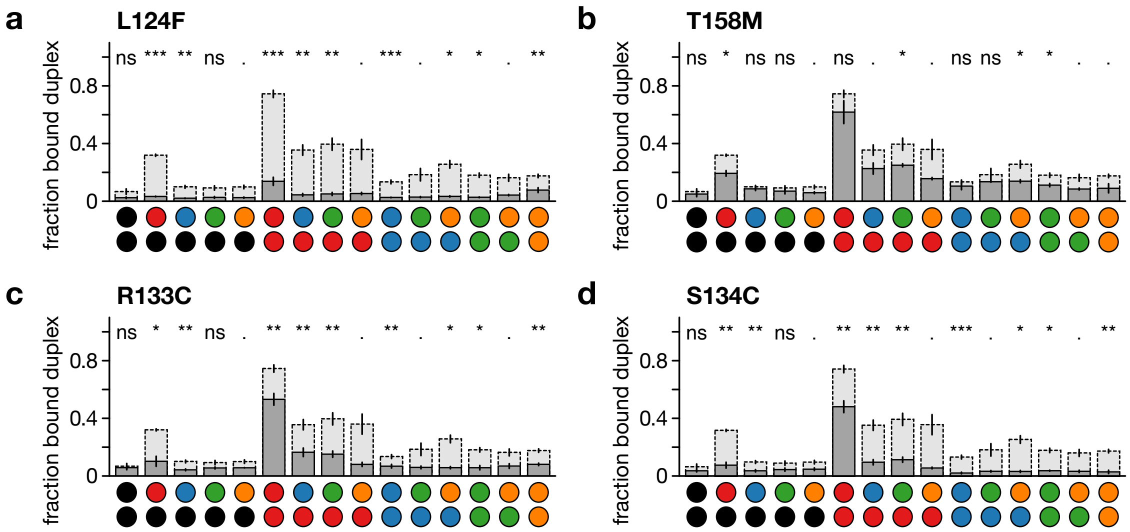

Supplementary Figure 10. Altered selectivity profiles of MeCP2 Rett mutants at low protein concentration. EMSA selectivity measurements of four Rett mutants in contrast to wildtype human MeCP2[90–181] (shown as light gray bars; see Supplementary Figure 5) at 128 nM MBD protein concentration. (**a**) In contrast to MeCP2[90–181]L124F (see Supplementary Figure 6); (**b**) In contrast to MeCP2[90–181]T158M (see Supplementary Figure 9); (**c**) In contrast to MeCP2[90–181]R133C (see Supplementary Figure 7); (**d**) In contrast to MeCP2[90–181]S134C (see Supplementary Figure 9). Duplex configurations in color code with the top row representing unlabeled strand and the bottom row the labeled strand (Supplementary Table X9; black: 2'-deoxycytidine, red: 5-methyl-2'-deoxycytidine, blue: 5-hydroxymethyl-2'-deoxycytidine, green: 5-formyl-2'-deoxycytidine, orange: 5-carboxy-2'-deoxycytidine). Error bars indicate standard error of the mean with two-sided Student's *t*-test against the fraction of bound mC/mC duplex (dark gray); false-discovery rate was controlled using the Benjamini-Hochberg procedure to correct the *p*-values for multiple comparisons (ns: *p* in (0.1, 1], . (0.05, 0.1], * (0.01, 0.05], ** (0.001, 0.01], *** [0, 0.001]).

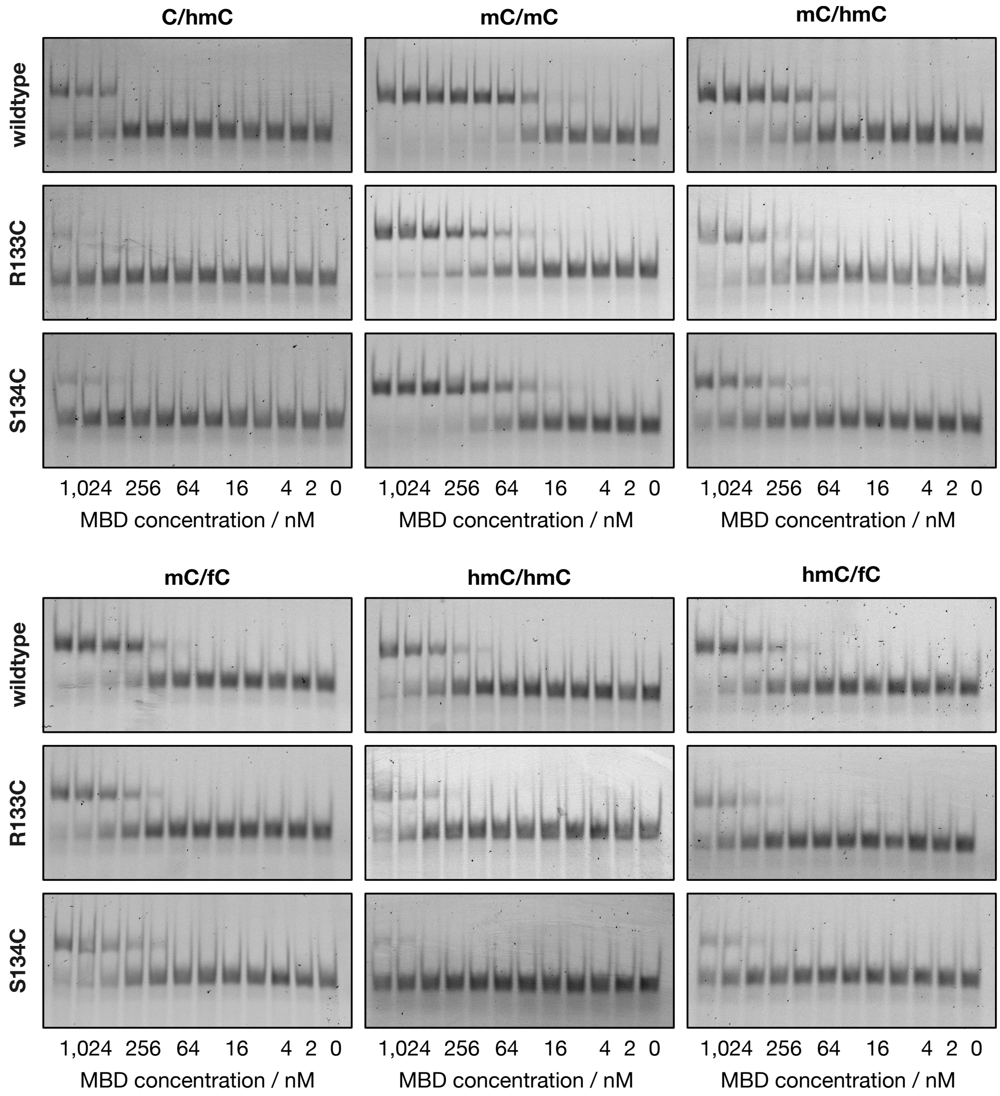

Supplementary Figure 11. Affinity measurements of MeCP2 Rett mutants (Representative EMSA gel images). EMSA gel images of a dilution series of MBD proteins at 2,048, 1,024, 512, 256, 128, 64, 32, 16, 8, 4, 2, and 0 nM with 2 nM of the labelled DNA duplexes containing the indicated 2'-deoxycytidine modifications.

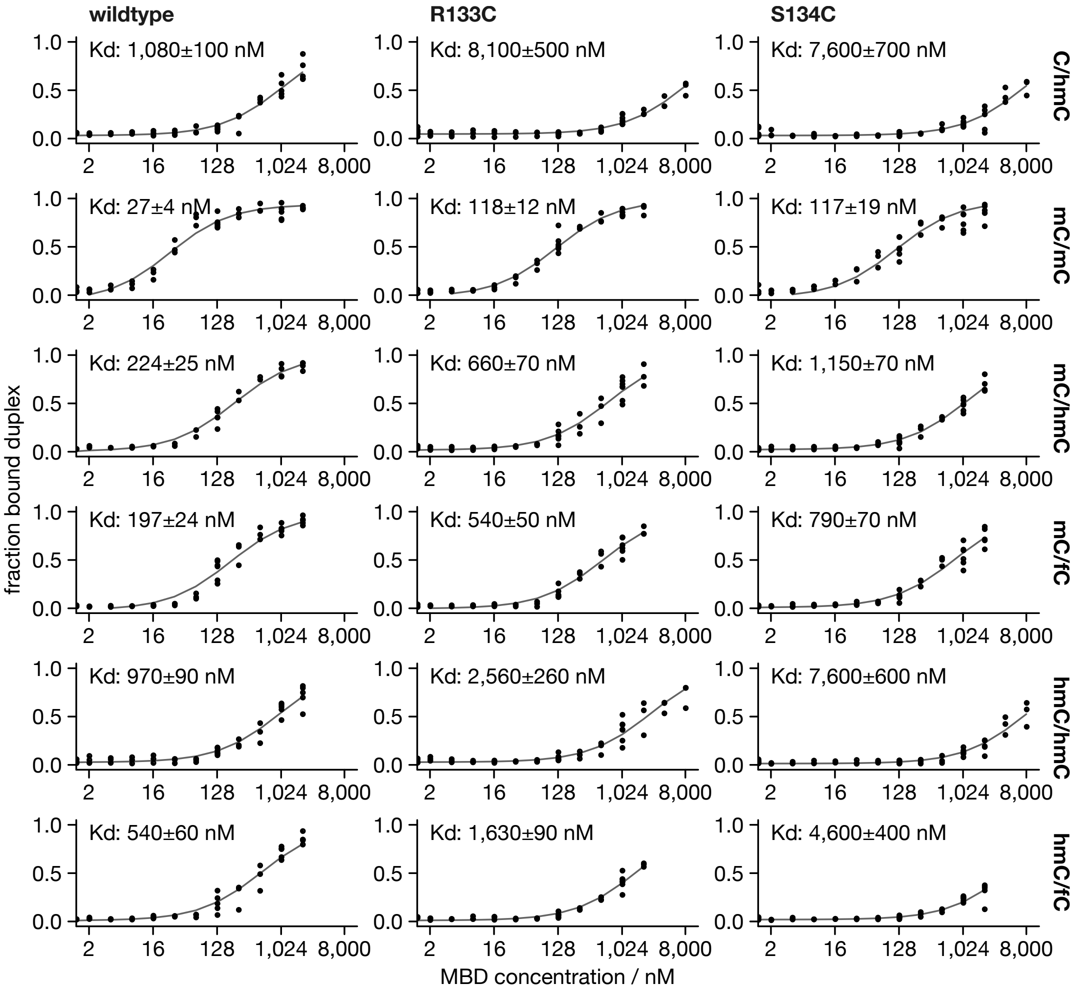

Supplementary Figure 12. Affinity measurements of MeCP2 Rett mutants (Fitted curves). Fitted fraction of bound labelled DNA duplex (see Supplementary Figure 11) as a function of MBD protein concentration (gray curves); non-linear least-square fit using the Levenberg-Marquardt algorithm (see Supplementary Table 6 and Methods for details). Best estimate of the dissociation constant K_d with standard error of mean given.

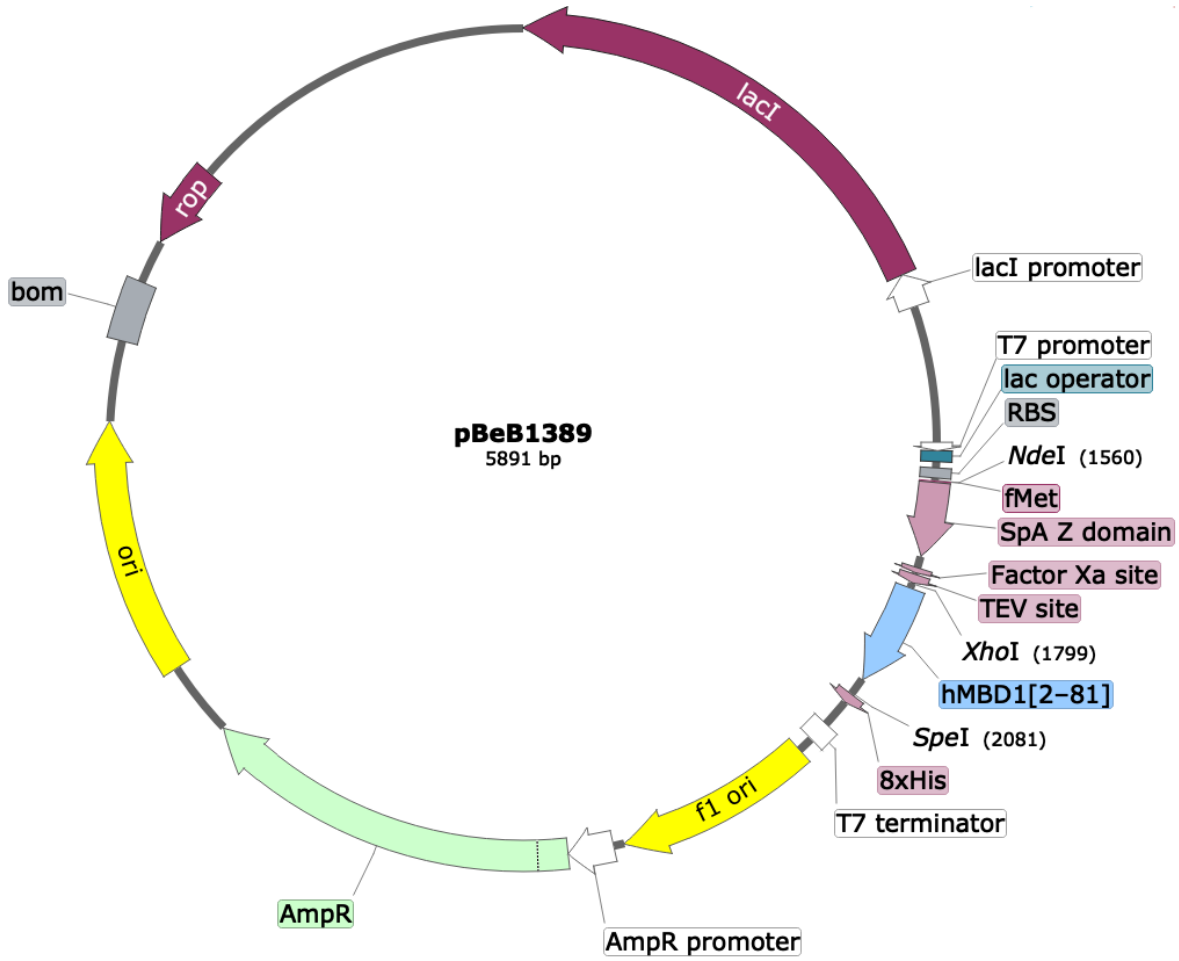

Supplementary Figure 13. Sample plasmid map of pBeB1380 with hMBD1[2–81]. The fusion protein translates into a 20 kDa fusion protein as MDNKFNKEQQNAFYEILHLPNLNEEQRNAFIQSLKDDPSQSANLLAEAKKLNDAQAPKVDAGSGSGSIEGRLENLYFQSLEAEDWLDCPALGPGWKRREVFRKSGATCGRSDTYYQSPTGDRIRSKVELTRYLGPACDLTLFDFKQGILCYPAPKAHPVAVTSHHHHHHHH*. Figure created with SnapGene software (GSL Biotech).

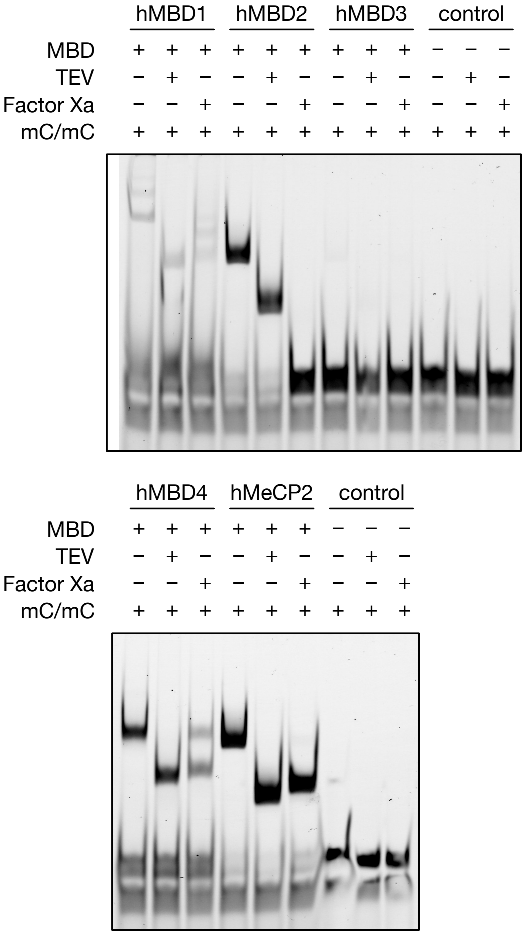

Supplementary Figure 14. Effect of TEV cleavage on SpA(Z)-MBD fusion proteins. EMSA at 4 nM labelled DNA duplex (with excess labelled strand, lowest immobile band) containing an mC/mC modified CpG with the indicated SpA(Z)-MBD fusion proteins at 1 µM MBD concentration (except for hMBD1, which was re-expressed for all other assays in this study).

Supplementary Tables

Supplementary Table 1. Plasmids.

**pBeB1380**	pET(21d)-SpA(Z)-[Factor Xa]-[TEV]-6xHis
**pBeB1389**	pBeB1380-hMBD1[2–81]-8xHis
**pBeB1390**	pBeB1380-hMBD2[146–225]-8xHis
**pBeB1391**	pBeB1380-hMBD3[2–81]-8xHis
**pBeB1392**	pBeB1380-hMBD4[76-167]-8xHis
**pBeB1393**	pBeB1380-hMeCP2[90–181]-8xHis
**pDaH1642**	pBeB1380-hMeCP2[90–181]S134C-8xHis
**pDaH1643**	pBeB1380-hMeCP2[90–181]L124F-8xHis
**pDaH1644**	pBeB1380-hMeCP2[90–181]R133C-8xHis
**pDaH1645**	pBeB1380-hMeCP2[90–181]T158M-8xHis

Supplementary Table 2. Oligodeoxynucleotides for plasmid construction.

pBeB1380 (pET(21d)-SpA(Z)-[Factor Xa]-[TEV]-6xHis)

**o2872_fwd**	ctttaagaaggagatatacatatgGACAACAAATTCAACAAAGAACAACAAAACGC
**o2873_rev**	agtggtggtggtggtggtgctcgagagactgaaaataaagattttcgagccttccctcgatagaaccactgcca<br>gatcccgcgtc

pBeB1389 (hMBD1[2–81])

**o2878**	GCAGAAGACTGGTTGGACTGTCCAGCTTTAGGTCCAGGTTGGAAACGCCGTGAGGTGTTTCGTAAGTCTGGTGC<br>AACGTGCGGTCGCTCCGATACCTACTACCAGTCACCTACCGGTGACCGCATTCGCTCTAAGGTAGAATTGACCC<br>GCTATCTTGGGCCGGCCTGCGATCTGACCTTATTCGATTTCAAACAGGGTATCTTGTGTTACCCCGCGCCCAAA<br>GCGCATCCCGTAGCAGTG
**o2879_fwd**	aaatctttattttcagtctctcgagGCAGAAGACTGGTTGGACTG
**o2880_rev**	gtggtggtggtggtggtgactagtCACTGCTACGGGATGCG

pBeB1390 (hMBD2[146–225])

**o2881**	TCAGGCAAACGTATGGATTGCCCCGCACTGCCTCCCGGATGGAAAAAGGAGGAGGTAATTCGCAAGTCAGGCCT<br>TAGTGCCGGCAAATCCGATGTATATTATTTCTCACCTAGTGGCAAGAAGTTTCGCTCAAAACCACAGTTAGCAC<br>GTTATTTGGGTAATACGGTTGACTTGTCCTCGTTCGATTTCCGTACCGGTAAAATGATGCCATCGAAATTACAG<br>AAGAATAAGCAACGCTTG
**o2882_fwd**	aaatctttattttcagtctctcgagTCAGGCAAACGTATGGATTG
**o2883_rev**	gtggtggtggtggtggtgactagtCAAGCGTTGCTTATTCTTC

pBeB1391 (hMBD3[2–81])

**o2884**	GAACGCAAACGCTGGGAATGCCCAGCTTTACCTCAAGGATGGGAACGCGAGGAGGTTCCTCGCCGCTCAGGCCTTTCCGCCGGCCATCGTGACGTATTTTACTACTCGCCTTCGGGAAAGAAATTCCGCTCAAAACCACAGTTAGCGCGTTACCTGGGAGGATCTATGGATTTGTCTACCTTCGACTTTCGCACCGGTAAAATGCTGATGAGCAAGATGAATAAATCTCGTCAACGCGTG
**o2885_fwd**	aaatctttattttcagtctctcgagGAACGCAAACGCTGGGAATG
**o2886_rev**	gtggtggtggtggtggtgactagtCACGCGTTGACGAGATTTATTC

pBeB1392 (hMBD4[76–167])

**o2884**	GCGACCGCAGGTACAGAGTGCCGTAAGTCAGTTCCCTGTGGGTGGGAGCGCGTTGTCAAGCAACGCTTGTTTGGTAAAACAGCCGGCCGTTTCGATGTTTACTTTATCTCGCCGCAAGGCCTAAAGTTCCGTTCCAAGTCATCACTTGCTAACTATTTACACAAAAACGGTGAAACCTCCCTTAAACCGGAAGACTTTGACTTCACTGTGTTAAGCAAGCGTGGTATCAAAAGCCGCTACAAGGACTGCTCAATGGCAGCATTAACATCCCATCTC
**o2885_fwd**	aaatctttattttcagtctctcgagGCGACCGCAGGTACAGAG
**o2886_rev**	gtggtggtggtggtggtgactagtGAGATGGGATGTTAATGCTGCC

pBeB1393 (hMeCP2[90–181])

**o2890**	GATCGTGGTCCTATGTATGATGATCCAACACTTCCTGAGGGCTGGACCCGTAAATTGAAGCAACGCAAATCCGGTCGCAGCGCCGGCAAGTATGATGTGTATCTTATCAATCCCCAAGGGAAGGCATTTCGCAGTAAGGTGGAGTTGATCGCATACTTCGAGAAAGTGGGTGATACATCTCTGGACCCTAATGACTTCGATTTTACCGTAACTGGGCGTGGAAGCCCTTCGCGTCGCGAGCAGAAACCCCCTAAAAAGCCAAAATCACCCAAAGCT
**o2891_fwd**	aaatctttattttcagtctctcgagGATCGTGGTCCTATGTATG
**o2892_rev**	gtggtggtggtggtggtgactagtAGCTTTGGGTGATTTTGG

pDaH1642 (hMeCP2[90–181] variant S134C)

**o3118_fwd**	aagggaaggcatttcgcTgtaaggtggagttgatc
**o3119_rev**	gatcaactccaccttacAgcgaaatgccttccctt

pDaH1642 (hMeCP2[90–181] variant L124F)

**o3120_fwd**	cggcaagtatgatgtgtatTttatcaatccccaagggaa
**o3121_rev**	ttcccttggggattgataaAatacacatcatacttgccg

pDaH1642 (hMeCP2[90–181] variant R133C)

**o3122_fwd**	ccccaagggaaggcatttTgcagtaaggtgg
**o3123_rev**	ccaccttactgcAaaatgccttcccttgggg

pDaH1642 (hMeCP2[90–181] variant T158M)

**o3124_fwd**	cctaatgacttcgattttaccgtaaTGgggcgtggaagcc
**o3125_rev**	ggcttccacgcccCAttacggtaaaatcgaagtcattagg

Supplementary Table 3. MBD protein sequences.

hMBD1[2–81] (CCDS59318.1)

AEDWLDCPALGPGWKRREVFRKSGATCGRSDTYYQSPTGDRIRSKVELTRYLGPACDLTLFDFKQGILC
YPAPKAHPVAV

hMBD2[146–225] (CCDS11953.1)

SGKRMDCPALPPGWKKEEVIRKSGLSAGKSDVYYFSPSGKKFRSKPQLARYLGNTVDLSSFDFRTGKM
MPSKLQKNKQRL

hMBD3[2–81] (CCDS12072.1)

ERKRWECPALPQGWEREEVPRRSGLSAGHRDVFYYSPSGKKFRSKPQLARYLGGSMDLSTFDFRTGK
MLMSKMNKSRQRV

hMBD4[76–167] (CCDS3058.1)

ATAGTECRKSVPCGWERVVKQRLFGKTAGRFDVYFISPQGLKFRSKSSLANYLHKNGETSLKPEDFDFT
VLSKRGIKSRYKDCSMAALTSHL

hMeCP2[90–181] (CCDS14741.1)

DRGPMYDDPTLPEGWTRKLKQRKSGRSAGKYDVYLINPQGKAFRSKVELIAYFEKVGDTSLDPNDFDFT
VTGRGSPSRREQKPPKKPKSPKA

Supplementary Table 4. Oligodeoxynucleotides (ODNs) for EMSA.

**o2968**	AAAAAAAAAAAAAAAAAAAAAAAA	
**o2969**	TTTTTTTTTTTTTTTTTTTTTTTT	
**o2903**	AAAAAAAAAAACGAAAAAAAAAAA	
**o2904**	TTTTTTTTTTTCGTTTTTTTTTTT	
**o2906**	[6-FAM]AAAAAAAAAAACGAAAAAAAAAAA	
**o2967**	[6-FAM]AAAAAAAAAAA[X]GAAAAAAAAAAA	X = 5-methyl-2’-deoxycytidine
**o2909**	TTTTTTTTTTT[X]GTTTTTTTTTTT	X = 5-methyl-2’-deoxycytidine
**o3115**	[6-FAM]AAAAAAAAAAA[X]GAAAAAAAAAAA	X = 5-hydroxymethyl-2’-deoxycytidine
**o3112**	TTTTTTTTTTT[X]GTTTTTTTTTTT	X = 5-hydroxymethyl-2’-deoxycytidine
**o3116**	[6-FAM]AAAAAAAAAAA[X]GAAAAAAAAAAA	X = 5-formyl-2’-deoxycytidine
**o3113**	TTTTTTTTTTT[X]GTTTTTTTTTTT	X = 5-formyl-2’-deoxycytidine
**o3117**	[6-FAM]AAAAAAAAAAA[X]GAAAAAAAAAAA	X = 5-carboxy-2’-deoxycytidine
**o3114**	TTTTTTTTTTT[X]GTTTTTTTTTTT	X = 5-carboxy-2’-deoxycytidine

Supplementary Table 5. ODN pairs for EMSA. See Supplementary Table X8 for ODN sequences.

**Probe**	**6-FAM labeled strand**		**unlabeled strand**	
binding trap	o2968	none	o2969	none
p01	o2906	C	o2904	C
p02	o2967	mC	o2904	C
p03	o3115	hmC	o2904	C
p04	o3116	fC	o2904	C
p05	o3117	caC	o2904	C
p06	o2967	mC	o2909	mC
p07	o3115	hmC	o2909	mC
p08	o3116	fC	o2909	mC
p09	o3117	caC	o2909	mC
p10	o3115	hmC	o3112	hmC
p11	o3116	fC	o3112	hmC
p12	o3117	caC	o3112	hmC
p13	o3116	fC	o3113	fC
p14	o3117	caC	o3113	fC
p15	o3117	caC	o3114	caC

Supplementary Table 6. Fit statistics. Summary statistic of fits shown in Supplementary Figure 12 and Figure 3g–i. logLik: $\log_{10}$-likelihood; AIC: Akaike information criterion; BIC: Bayesian information criterion; df: degrees of freedom; CI: confidence interval.

Assay			Non-least square fit						
Protein	Probe	Gels	Sigma	Convergent	logLik	AIC	BIC	Deviance	Residual df
wildtype	p03	3	0.0591	TRUE	61.64	-117.28	-112.00	0.14	41
wildtype	p06	3	0.0764	TRUE	50.58	-95.15	-89.87	0.24	41
wildtype*	p06	3	0.0595	TRUE	62.41	-114.82	-106.01	0.14	39
wildtype	p07	2	0.0514	TRUE	49.03	-92.06	-87.76	0.08	29
wildtype	p08	3	0.0679	TRUE	55.67	-105.33	-100.05	0.19	41
wildtype	p10	3	0.0582	TRUE	62.33	-118.66	-113.37	0.14	41
wildtype	p11	3	0.0681	TRUE	54.25	-102.49	-97.28	0.19	40
R133C	p03	6	0.0369	TRUE	98.82	-191.64	-185.78	0.07	50
R133C	p06	3	0.0548	TRUE	64.87	-123.74	-118.45	0.12	41
R133C	p07	3	0.0686	TRUE	55.24	-104.48	-99.20	0.19	41
R133C	p08	3	0.0535	TRUE	65.88	-125.75	-120.47	0.12	41
R133C	p10	6	0.0683	TRUE	66.81	-127.61	-121.76	0.23	50
R133C	p11	3	0.0359	TRUE	83.07	-160.15	-154.86	0.05	41
S134C	p03	6	0.0520	TRUE	82.51	-159.01	-153.10	0.14	51
S134C	p06	3	0.0885	TRUE	45.28	-84.56	-79.20	0.33	42
S134C	p07	3	0.0405	TRUE	79.66	-153.32	-147.97	0.07	42
S134C	p08	3	0.0599	TRUE	62.49	-118.98	-113.63	0.15	42
S134C	p10	6	0.0443	TRUE	90.97	-175.94	-170.03	0.10	51
S134C	p11	3	0.0373	TRUE	83.25	-160.51	-155.15	0.06	42

Assay			Estimates			95% CI		Relative estimates		95% CI	
Protein	Probe	Gels	Kd	SEM	statistic	lower	upper	Kd	SEM	lower	upper
wildtype	p03	3	1078.88	100.93	10.69	897.76	1295.48	40.57	10.54	25.65	64.05
wildtype	p06	3	26.59	3.85	6.91	20.23	35.00	1.00	0.37	0.58	1.73
wildtype*	p06	3	29.29	2.27	12.89	25.11	34.23	1.00	0.62	0.73	1.36
wildtype	p07	2	224.18	24.61	9.11	181.56	277.39	8.43	2.22	5.19	13.71
wildtype	p08	3	197.00	24.04	8.20	157.68	246.53	7.41	1.95	4.51	12.19
wildtype	p10	3	967.10	89.64	10.79	806.38	1157.97	36.36	9.45	23.04	57.25
wildtype	p11	3	541.33	62.73	8.63	436.90	667.67	20.35	5.30	12.48	33.01
R133C	p03	6	8113.50	526.58	15.41	7115.53	9292.79	68.78	5.84	49.20	96.48
R133C	p06	3	117.97	11.79	10.01	96.31	144.62	1.00	0.12	0.67	1.50
R133C	p07	3	660.22	72.89	9.06	531.87	816.15	5.60	0.48	3.68	8.47
R133C	p08	3	543.14	47.98	11.32	456.78	644.06	4.60	0.40	3.16	6.69
R133C	p10	6	2555.93	261.19	9.79	2066.16	3173.48	21.67	1.84	14.29	32.95
R133C	p11	3	1627.88	93.44	17.42	1454.67	1825.17	13.80	1.18	10.06	18.95
S134C	p03	6	7569.67	660.58	11.46	6387.07	9043.24	64.96	3.51	36.93	114.40
S134C	p06	3	116.53	18.54	6.29	79.05	172.96	1.00	0.08	0.46	2.19
S134C	p07	3	1149.66	71.64	16.05	1014.80	1301.84	9.87	0.55	5.87	16.47
S134C	p08	3	790.04	74.95	10.54	651.16	954.99	6.78	0.38	3.76	12.08
S134C	p10	6	7627.15	568.44	13.42	6604.58	8859.12	65.45	3.53	38.19	112.07
S134C	p11	3	4644.37	354.75	13.09	3994.79	5460.87	39.86	2.15	23.10	69.08

* Binding of MeCP2 to probe p06 (mC/mC) is more likely to follow a binding model with Hill slope (with $h = 1.91 \pm 0.24$), which does however not significantly impact the Kd estimate (at a significance level of 0.05).
